# Techniques to assess reproductive status in adult female American alligators (*Alligator mississippiensis*) using laparoscopic examination

**DOI:** 10.1371/journal.pone.0287140

**Published:** 2023-11-15

**Authors:** Mark Flint, Jaylene B. Flint, Jeffrey D. Miller

**Affiliations:** 1 One Welfare and Sustainability Center, Department of Veterinary Preventive Medicine, College of Veterinary Medicine, The Ohio State University, Columbus, OH, United States of America; 2 Biological, Research and Education Consultants, Missoula, MT, United States of America; AIIMS: All India Institute of Medical Sciences, INDIA

## Abstract

This protocol describes a minimally invasive surgical technique and approach to successfully examine the gonads of live female American alligators as part of a reproductive examination used in conservation medicine and biology. Best practices are based on examination of over 80 American alligators in the last two years adapting principles derived from other reptilian megafauna species. This protocol is designed for appropriately qualified veterinarians and biologists working in the field. We show likely reproductive tract presentations with respect to breeding status and environmental cues to help guide interpretation of observations. The laparoscopic approach and findings presented here provide tools to safely clinically examine animals in a welfare-oriented way that will advance our understanding of crocodilian reproduction. This technique has not previously been described in this species.

## Introduction

The American alligator (*Alligator mississippiensis*) was listed as endangered in 1967 and was one of the first species protected under the Endangered Species Act of 1973. A conservation success story, it was removed from the list in 1987, but has been managed to ensure survival since. Further, approximately 1.5M crocodilian skins are harvested and sold annually around the world, of which American alligators provide one third of this global market, making it a valuable commodity [1]. Consequently, it is important to understand population health for ongoing species management. The fitness response of a free-ranging American alligator population can be assessed using multiple techniques. For example, population size can be estimated through surveys conducted over time and sex ratios within size classes can be determined through assessing testosterone levels in the blood or via digitally examining the copulatory organ inside the vent [2–4]. Total eggs laid and hatched each year can be estimated by performing egg counts of nests after the nesting season [5, 6] to give a proxy of population growth. Minimally-invasive breeding indicators can be determined through blood sampling for hormone ratios.

These approaches follow sound population biology techniques, but do not utilize a unique anatomical feature of some reptile species, including American alligators, to predict the reproductive capacity and periodicity of individuals within a population. The reproductive tracts of female sea turtles, freshwater turtles, as well as crocodiles and alligators have an approximate three-year “record” of reproduction that can be seen on examination [7–10]. Direct visualization of reproductive organs via laparotomies or laparoscopy allows establishment of the sex and maturity of the individual animal, as well as its reproductive status. Although laparotomies have been used on female Nile crocodiles for the examination of their ovaries [11] and Australian Freshwater crocodiles [10], the application of laparoscopy is the better choice for examination of reproductive structures and determination of status because it is a minimally invasive technique and allows superior close-up access to the structures. The more readily accessed and assessed presence of developing, mature, and regressing follicles along with formation and resolution of atretic follicles can be used to interpret whether the reptile bred last year or the year before, or if it will breed this year or potentially next year [9, 12]. These factors make this approach a useful, accurate, minimally invasive and welfare-oriented field technique.

The examination of the reproductive organs via laparoscopy requires surgery and interpretation. Herein we describe the surgical considerations and approach to abdominal laparoscopy for reproductive tract examination. In addition, we provide a preliminary interpretation of reproductive features in adult female American alligators.

## Materials and methods

All work was conducted following humane welfare and ethical standards overseen by an American College of Animal Welfare Board Certified Veterinary Specialist under The Ohio State University’s IACUC Protocol #2019A00000144.

The protocol described in this peer-reviewed article is published on protocols.io, https://www.protocols.io/view/step-by-step-protocol-for-the-laparoscopic-examina-36wgqj8r5vk5/v1 and is included for printing as S1 File with this article.

### Surgical approach

#### General health assessments

Before any animal is examined using laparoscopy, it should be medically assessed to ensure it is fit and able to have a minimally invasive procedure conducted. Examination should include visual inspection of body condition score, activity level, and overt signs of illness or trauma [13, 14].

#### Stress myopathy

Because there is the potential for stress myopathy and/or stress-induced mortality as a result of capture in many species, including reptiles [15, 16], alligators are captured and handled following recommended procedures devised through consultation with commercial hunters, various state wildlife officers, and the authors own experiences. If the animal does not pass a general health assessment, it should not be subjected to laparoscopic examination because of the increased risk of stress induced morbidity or mortality. If at any stage during the capture or surgical procedure the animal becomes unresponsive, all activities should stop immediately and the animal should be placed in a shaded area and monitored for recovery. Intervention may be required. All examined animals should be clearly marked (with a grease marker or spray paint) prior to release, placed near water and monitored until they voluntarily enter the water. Marking the animal allows ongoing distant monitoring of the alligator post release behavior and helps reduce the likelihood of recapture (and additional stress).

#### Anesthesia and local anesthetic

When indicated, sedation to light planes of anesthesia should be used to minimize stress and risk during laparoscopic examination. Commonly reported crocodilian sedatives are either ketamine or xylazine [17]. Consideration should be given to protracted recovery time using anesthetics in reptiles and the environment into which the animal is being released (e.g., heat, cannibalistic cohorts).

Local anesthetic at the site of incision for the laparoscopic examination should always be used. We use a small dose (approximately 10mg) of lidocaine hydrochloride 2% solution delivered subcutaneously via a 22G needle.

#### Positioning

In order to best visualize the reproductive tract, immobilizing the alligator upside down on a solid structure such as a ladder and tilting them head-down on a 45–60° angle encourages the loops of intestine to move cranially and expose the caudo-dorsally located reproductive tract. This position does not impede respiration but is known to induce a relaxed sedated-like state where the animal can be easily roused, if required.

#### Landmarks

**Fig 1:**
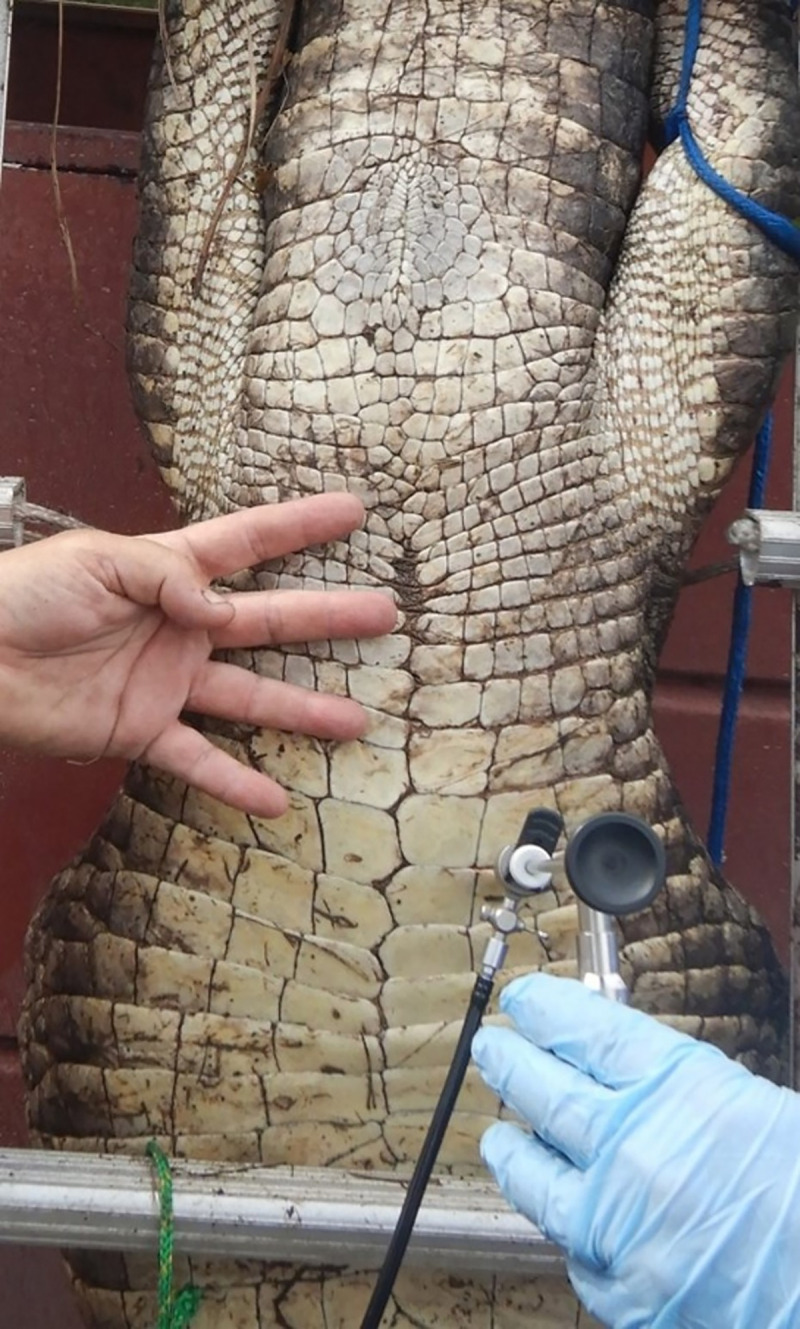
Surgical approach to gain best access to the reproductive organs. At least 1 to 3 scales cranial of the cranial pelvic girdle and 2 to 3 scales lateral of midline.

Crocodile gonads should be accessed using a lateral-midline approach. By contrast, we have found alligators are best examined on the alligator’s right-hand side (your right when upside down and ventral surface facing you) via a small 1.0 cm incision made between the scales located 2–3 full scales below the most cranial aspect of the ventral pelvic girdle and 2–3 scale rows lateral of midline (Fig 1).

#### Hazards

Hazards to avoid include the abdominal wall which is muscular and layered with mesentery. Entry via trocar and cannula should be perpendicular to the wall of the body cavity. The positioning must be maintained to penetrate the body cavity. If the surgical entry site is too far lateral or if the angle of entry is not maintained the trocar and cannula may not penetrate successfully. Also, alligators have gastric ribs (gastralia) that run most of the length of the abdomen. Incise medial to these ribs and be aware entering too closely to the pelvis or the ribs, because both will restrict the ability to rotate the laparoscope.

#### Laparoscopic equipment used

We use a Karl Storz 5 mm x 29 cm 30° angle lens laparoscope fitted with a battery-operated portable light source. The laparoscope enters inside either an 8.5 or 20cm long by 6 mm wide trocar and cannula set. The longer length may be required to access the abdominal cavity containing the reproductive tract in larger animals.

All instruments are kept sterile in an alcohol-based cleaning solution within closed containers until being used and then returned to the container in preparation for subsequent use.

Cell phones may be used to take video or photographs of any findings. This may be aided by using easily available cell-phone microscope holders. Some quick modifications allow the eyepieces of the laparoscope and cell-phone holder to seat easily (wound bandage around the scope eyepiece to make it bulkier) giving a quick connection and stable image.

#### Incision and accessing the reproductive tract

As outlined, the optimal location for the incision is 2 to 3 scale rows cranial of the cranial-most point of the pelvic girdle and 2 to 3 scale rows lateral of midline on the animal’s right side. The surgical area should be clean of all algae and dirt using a fingernail brush, then the area should be scrubbed again with surgical scrub, such as chlorhexidine, before being washed with alcohol. Local anesthetic should be injected advancing along the site of incision. When the anesthetic has activated, the incision should be approximately 1cm long penetrating the skin layers and subcutaneous membranous tissue. We find a Size 11 or 15 scalpel gives good control and access. Incise between the scales. Do not cut through the scales. Once the incision has been made, insert the cannula and trocar blunt dissecting tissue. If there is resistance, gently advance the trocar through the cannula to pierce the tissue, re-sheath the trocar and continue until the cannula is in place. Frequently remove the trocar when advancing and visualize placement with the laparoscope to minimize the risk of going between muscle layers or puncturing intestine. Sometimes there is a final layer of mesentery surrounding the reproductive tract that needs to be penetrated. Once the cannula is in place, gently insufflate the abdomen. This can be achieved either by using a hand or foot operated pump to control the amount of air administered or via a medical grade pressurized air tank. Inflate sufficiently to help displace the intestines cranially and allow space to move the laparoscope. Do not overinflate because it may cause trapped air in dead spaces post-surgery or compress the diaphragm impeding respiration. After every one or two pumps or short bursts of air, reassess using the laparoscope to determine if the gonads can be seen. In general, only a few pumps/puffs of air are required and the abdomen can be seen to rise as it inflates.

#### Examination of reproductive tract

The reproductive tract of females can be examined either cranially to caudally or vice versa. However, the entire tract of at least one side must be examined because development progresses from caudal to cranial and reproductive status is determined based on multiple factors. Not visualizing as much of the tract as possible may lead to an incorrect determination of breeding status. This may require rotating the animal, using your laparoscope to move intestine, or adding a small amount of additional air to expose structures. All structures should be measured to quantify what is seen. Measurement should be made after the field of view of the laparoscope has been calibrated against a reliable metric ruler. The size of a structure can be measured by touching the structure with the tip of the scope.

#### Exiting and closure

Once examination is complete, remove the laparoscope and replace it into the alcohol-based cleaning solution. While exerting moderate pressure on the abdomen, open the cannula valve to expel all the air used to insufflate. When no more air can be removed, pull the cannula and place it into the cleaning solution. Do not release pressure on the abdomen because doing so may draw air back into the cavity. Using dissolving sutures, place a single full thickness mattress suture in the incision site to close the area. Sutures should be placed tight enough to ensure no air or liquid can enter once the animal is returned to the water.

#### Monitoring post procedure

The entire examination from scrubbing to suturing should take no longer than 10 minutes. Return the alligator to a normal resting position and remove all restraints except for the mouth tape until all handlers are safely behind some form of barrier. If sedation was required, keep the animal in a holding pen in a shaded area until it has fully recovered. If no sedation was required, mark the animal and place them several feet from the water source, allowing them to be observed walking, entering the water, and swimming away without impediment.

## Expected results

Using this technique, we were able to access, visualize, describe and interpret several structures outlined here. All alligators examined were adults and caught in northern Florida after vocalization (courtship) had begun for the season and 12 weeks prior to that season’s recorded nesting period.

### Interpretation of female reproductive structures

Interpretation of the reproductive status of mature females requires thorough examination of the ovary and oviduct, as well as consideration of the phase (early, mid, late) of reproduction season at the study location.

An immature ovary will contain numerous small (< 1–3 mm) previtellogenic follicles and may exhibit some additional follicles that are larger. However, the width of the flaccid oviduct will be less than 10–15 mm in the vicinity of the ovary.

**Fig 2:**
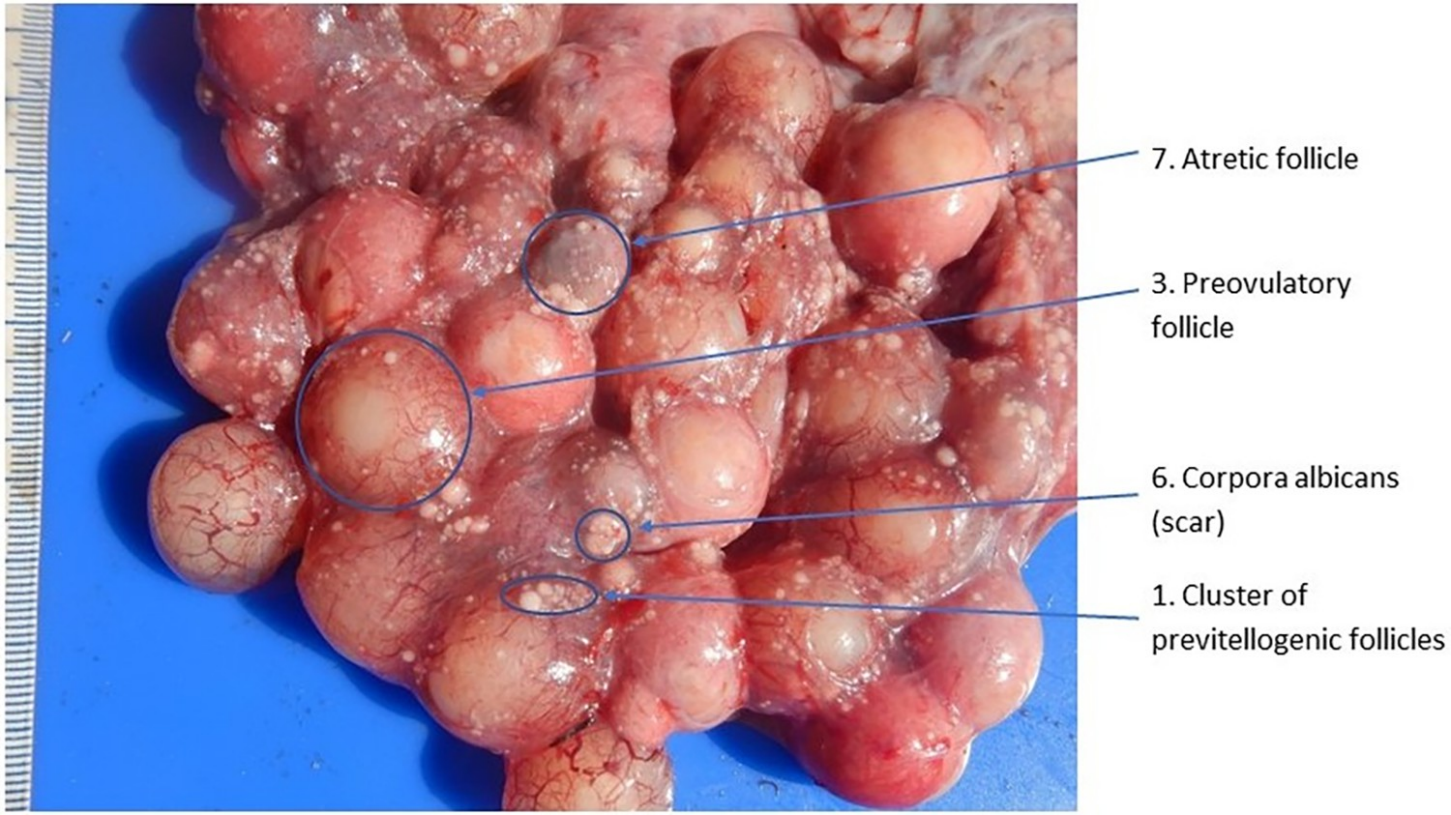
Externalized ovary of 2.68 m, 14 year old female alligator preparing to breed approximately 8 weeks prior to onset of nesting season, illustrating complex presentation of ovarian structures. (Numbers refer to descriptions in Table 1).

**Fig 3:**
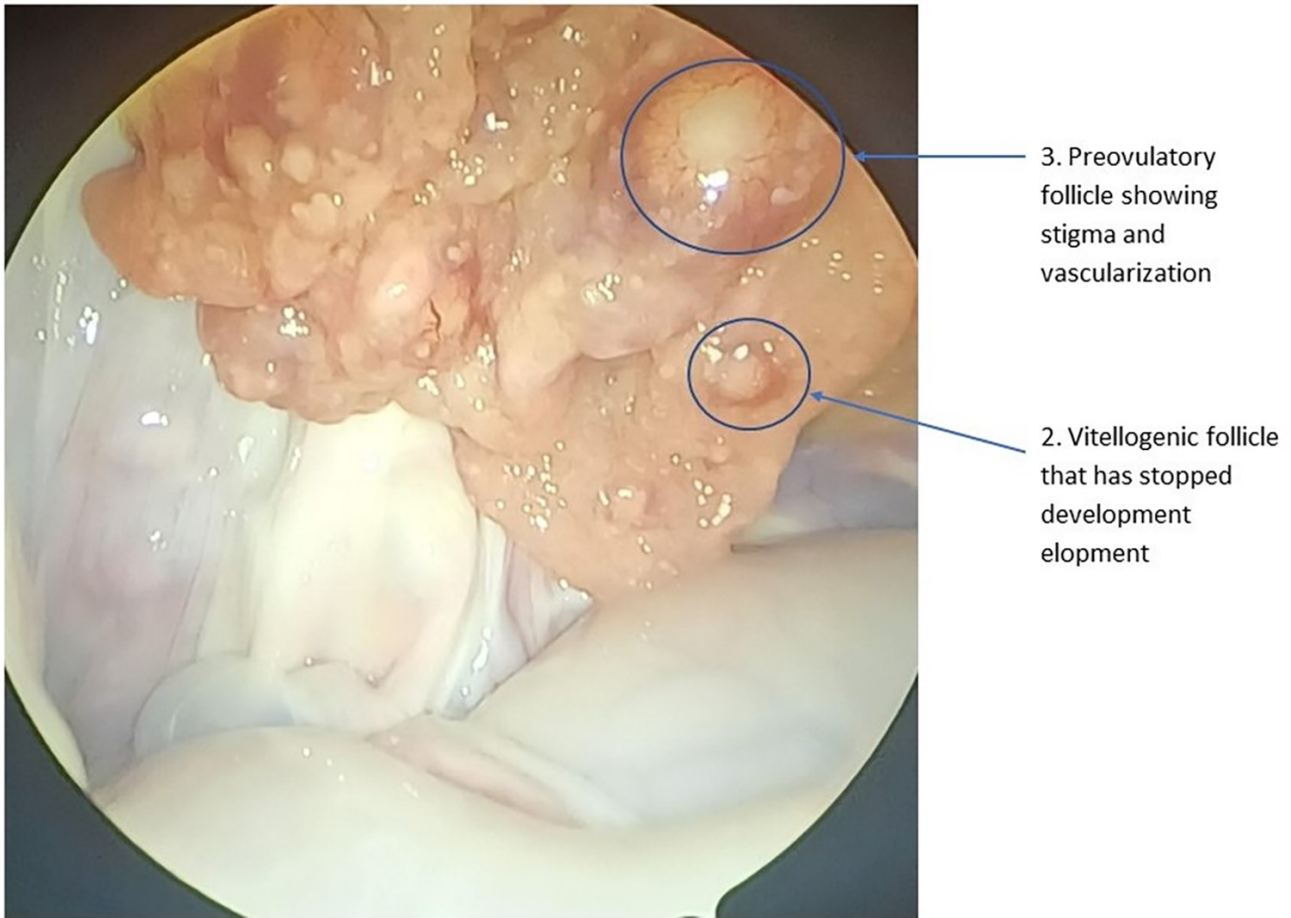
View through laparoscope of ovary of a 2.41 m 15 year old female preparing to breed this year demonstrating enlarged follicles, vesicles and expanded oviduct approximately 12 weeks before nesting season. Small follicles starting to show reaction to stimulation are also present. (Numbers refer to descriptions in Table 1).

In mature females, the expanded stroma of the ovary will support numerous small previtellogenic follicles with additional larger follicles that vary in size **(Table 1)**. Other structures, including corpus lutea, corpus albicans, and atretic follicles, may be visible depending on reproductive history **(Figs 2 and 3)**. The white, flaccid oviduct will be >25 mm wide in the vicinity of the ovary. In the adult female, the oviduct will be white and convoluted with transverse folding on its surface.

**Table 1:** Classification of follicular development prior to ovulation, as well as descriptions of corpus luteum, corpus albicans, and atretic follicles in ovaries of American Alligators as observed via laparoscopy. [See: Milnes [5], Lance [6], Guillette, Woodward [18], Uribe and Guillette [20] for descriptions of the reproductive cycle and/or histological descriptions].

Figure Label	Stage	Follicle: Description (size, color, comment)
1	Previtellogenic follicle	0.1–2 mm. Yellow to creamy-yellow. Numerous and distributed over compact stroma.
2	Vitellogenic follicle	3–20 mm. Yellow to creamy-yellow. Maybe more than one size class. Larger follicles exhibit obvious vascularization in stroma and the Stigma is indicated.
3	Preovulatory follicle	20–30 mm. Follicles contained in a richly vascularized stroma. Stigma clearly visible.
4	Corpus hemorrhagicum	Temporary structure formed when the capillaries of the stroma rupture during ovulation, resulting in a central blood clot.
5	Corpus luteum	20–25 mm. Pale-cream to pale-yellowish, collapsed cavity with irregular pursed shaped edge and a dark core. Size decreases with age to become white disc-shape with a depressed center.
6	Corpus albicans	<20 mm. White with collapsed cavity with condensed, darker core; center is pursed and stroma may support some capillaries, mostly near the margins. Size decreases as structure ages to become white disc-shape ring with a depressed center.
7	Atretic follicle	<30 mm. Orange to dark Reddish in colored, extensively vascularized. May have darker core with or without superficial blood vessels visible through stigma. Size decreases as structure ages.
8	Adult Oviduct	20–30 mm wide in the vicinity of the ovary. White, with transverse folds.

The corpus hemorrhagicum is formed when the capillaries of the ovarian stroma rupture during ovulation. The hemorrhage is normally slight and temporary. The resulting clot forms the dark center mass of the corpus luteum.

**Fig 4:**
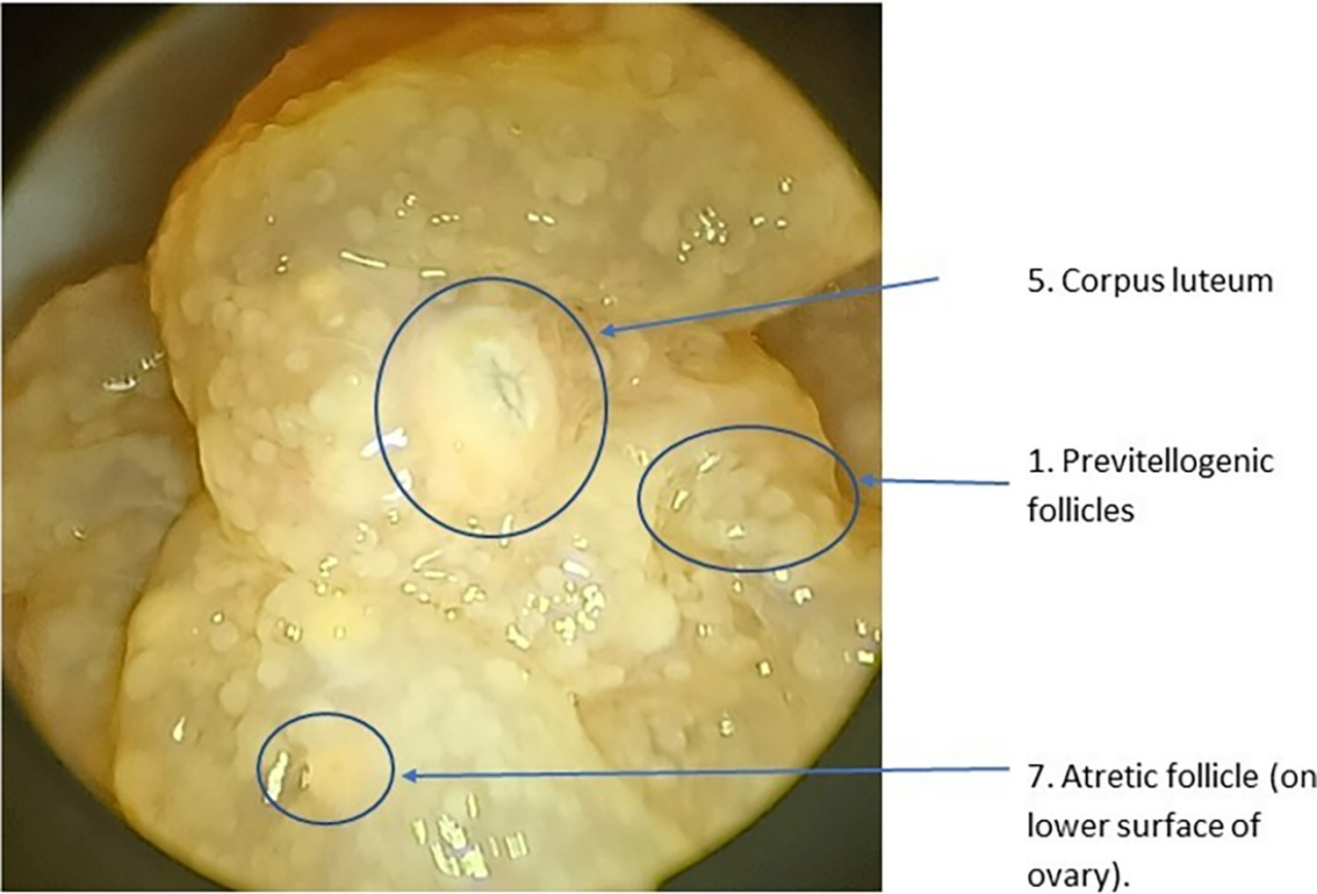
2.25 m 12 year old female who bred last year or the year before showing a follicular scar and inactive follicles 12 weeks before nesting season. (Numbers refer to descriptions in Table 1).

The cream/yellow-colored corpus luteum forms as the result of ovulation **(Fig 4).** The consolidated mass of the corpus luteum approximates the volume of the follicle when viewed shortly after ovulation. The volume of the corpora lutea decreases via luteolysis over a period of nine or more months following oviposition [18]. The area of the stigma is pursed, with a darker center.

The corpus albicans is the regressed form of the corpus luteum that has ceased hormone production **(Figs 1 and 4).** The degraded structure with its darker center will persist as a progressively smaller, white, avascular scar.

An atretic follicle appears orange to dark red-ish in color and is extensively vascularized surface. An atretic follicle may have a darker core with or without blood vessels being visible through stigma. Size decreases as the structure regresses to become a white disc-shape ring.

**Fig 5:**
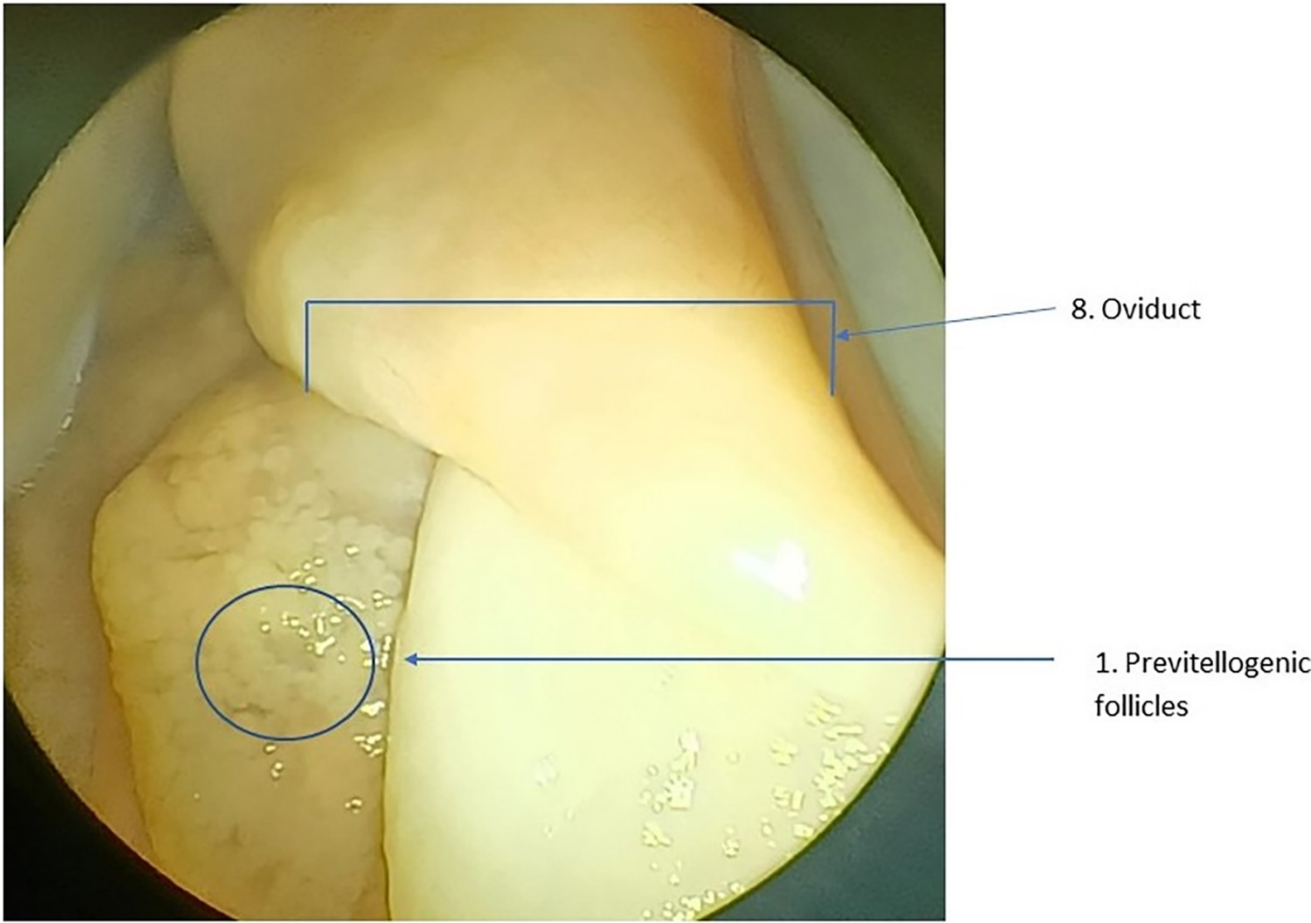
Quiescent ovary of a 2.29 m adult of unknown age who may breed next year showing small previtellogenic follicles and an expanded oviduct 12 weeks before nesting season. (Numbers refer to descriptions in Table 1).

The interpretation of reproductive status following examination of the ovary and oviduct must be tempered by the process of luteolysis. For example, early in the season (before ‘bellowing’ begins), the ovary may display numerous large corpora lutea and few or no enlarged follicles (**Fig 5**), that indicate it bred last year, or the ovary may display multiple larger vitellogenic follicles, along with smaller corpora albicantia and atretic follicles, indicating preparation to breed this year (**Figs 2 and 3**).

When multiple corpora lutea/albicantia are obvious in the stroma in spring, the female is unlikely to reproduce in the current state. The persistence of corpora lutea prevents breeding in the following reproductive season [18, 19]. Guillette, Woodward [19] reported detectable levels of vitellogenin in the plasma of about 40% of the female alligators sampled in April in Florida, which means that the only a portion of the population was preparing to breed. Our observations via laparoscopy on the presence of large corpora lutea/albicantia in the ovaries of alligators in the early spring (March to April) concur.

If the ovary is undergoing vitellogenesis, laparoscopy during the middle and late portions of the breeding season will show the presence of large vitellogenic follicles, as well as smaller corpora albicantia, and atretic follicles.

## Supporting information

S1 File Step-by-step protocol, also available on protocols.io. (PDF)
