# Supplementary material for: Techniques to assess reproductive status in adult female American alligators (Alligator mississippiensis) using laparoscopic examination
Source: PLoS One. 2023 Nov 15;18(11):e0287140. doi: 10.1371/journal.pone.0287140 (PMC10651014; doi:10.1371/journal.pone.0287140)
Supplement: S1 File — (PDF) [file pone.0287140.s001.pdf]

APR 17, 2023

Step by step protocol for the Laparoscopic examination of American alligators

Jaylene Flint[1], Mark Flint[1], Jeff Miller[1]

[1]The Ohio State University

Jaylene Flint
The Ohio State University

OPEN ACCESS

DOI: dx.doi.org/10.17504/protocols.io.36wgqj8r5vk5/v1

Protocol Citation: Jaylene Flint, Mark Flint, Jeff Miller 2023. Step by step protocol for the Laparoscopic examination of American alligators. **protocols.io** https://dx.doi.org/10.17504/protocols.io.36wgqj8r5vk5/v1

License: This is an open access protocol distributed under the terms of the Creative Commons Attribution License, which permits unrestricted use, distribution, and reproduction in any medium, provided the original author and source are credited

Protocol status: Working
We use this protocol and it's working

Created: Apr 17, 2023

Last Modified: Apr 17, 2023

PROTOCOL integer ID: 80642

ABSTRACT

This protocol describes a minimally invasive surgical technique and approach to successfully examine the gonads of live American alligators as part of a reproductive examination used in conservation medicine and biology.

MATERIALS

- Surgical platform that can be used to secure and position the animal
- Surgical scrub
- 70% alcohol
- Cleaning equipment
- Lidocaine 2% solution
- Needles and 22G syringes
- Karl Storz 5 mm x 29 cm 30° angle lens laparoscope fitted with a battery-operated portable light source and camera receptacle
- Camera
- 8.5cm and 20cm long by 6 mm wide trocar and cannula set
- Equipment sterilizers
- Suture kit including Size 11 or 15 scalpel blades and absorbable suture material
- Pump or low pressurized medical grade air cannister with connectors.

Surgical approach

1 Before any animal is examined using laparoscopy, it should be medically assessed to ensure it is fit and able to have a minimally invasive procedure conducted. Examination should include visual inspection of body condition score, activity level, and overt signs of illness or trauma.

2 When indicated, sedation to light planes of anesthesia should be used to minimize stress and risk during laparoscopic examination. Commonly reported crocodilian sedations are either ketamine or xylazine. Consideration should be given to protracted recovery time using anesthetics in reptiles and the environment into which the animal is being released (heat, cannibalistic cohorts).

3 Immobilize the alligator upside down on a solid structure such as a ladder and tilting them head-down on a 45-60° angle.

4 The surgical area should be clean of all algae and dirt using a fingernail brush then the area should be scrubbed again with surgical scrub, such as chlorhexidine, before being washed with alcohol.

5 Use a small dose (approximately 10mg) of lidocaine hydrochloride 2% solution delivered subcutaneously via a 22G needle at the site of incision for the laparoscopic examination. Local anesthetic should be injected advancing along the site of incision.

6 Using a size 11 or 15 scalpel blade, make a small 1.0 cm incision made between the scales at least one, and preferably 2-3, full scales below the most cranial aspect of the ventral pelvic girdle and 2-3 scale rows lateral of midline on the alligator’s right-hand side provides best access.

7 Insert the cannula and trocar, blunt dissecting tissue through the incision site. If there is resistance, gently advance the trocar through the cannula to pierce the tissue, re-sheath the trocar and continue until the cannula is in place.

8 Frequently remove the trocar when advancing and visualize placement with the laparoscope.

9 Once the cannula is in place, gently insufflate the abdomen by a hand or foot operated pump or

via a medical grade pressurized air tank. Inflate sufficiently to help displace the intestines cranially and allow space to move the laparoscope. Do not overinflate.

10 After every one or two pumps or short bursts of air, reassess using the laparoscope to determine if the gonads can be visualized. In general, only a few pumps/puffs of air are required and the abdomen can be seen to rise as it inflates.

11 Now the entire reproductive tract of males and females can be examined either cranially to caudally or vice versa.

12 After examination, remove the laparoscope and replace it into the alcohol-based cleaning solution.

13 Open the cannula valve to expel air by exerting moderate pressure on the abdomen.

14 When no more air can be removed, pull the cannula and place it into the cleaning solution. Do not release pressure on the abdomen.

15 Using dissolving sutures, place a single full thickness mattress suture in the incision site to close the area. Sutures should be placed tight enough to ensure no air or liquid can enter once the animal is returned to the water.

16 Return the alligator to a normal resting position and remove all restraints

17 Observe the animal is free of sedation and able to safely return to the water before releasing.
